# Diet modification reverses diastolic dysfunction in rats with heart failure and preserved ejection fraction

**DOI:** 10.1016/j.jmccpl.2023.100031

**Published:** 2023-01-04

**Authors:** Myung Yoon Kim, Isabelle Pellot, Catherine Bresee, Asma Nawaz, Mario Fournier, Jae Hyung Cho, Eugenio Cingolani

**Affiliations:** a Smidt Heart Institute, Cedars-Sinai Medical Center, United States of America; b Biostatistics and Bioinformatics Research Center, Cedars-Sinai Medical Center, Los Angeles, CA 90048, United States of America

**Keywords:** Heart failure with preserved ejection fraction, Diet, Blood pressure, Diastolic function, Hypertension, Ventricular arrhythmias

## Abstract

Dahl Salt-Sensitive (DSS) rats develop heart failure with preserved ejection fraction (HFpEF) when fed a high-salt (8 % NaCl) diet. Hypertension-induced inflammation and subsequent ventricular fibrosis are believed to underlie the development of HFpEF. We investigated the role of diet modification in the progression of HFpEF using male DSS rats, fed either a high-salt diet from7 weeks of age to induce HFpEF, ora normal-salt (0.3% NaCl) diet as controls. After echocardiographic confirmation of diastolic dysfunction at 14–15 weeks of age along with HF manifestations, the HFpEF rats were randomly assigned to either continue a high-salt diet or switch to a normal-salt diet for an additional 4 weeks. HFpEF rats with diet modification showed improved diastolic function (reduced E/E′ ratio in echocardiogram), increased functional capacity (increased treadmill exercise distance), and reduced pulmonary congestions (lung/body weight ratio), compared to high-salt-fed HFpEF rats. Systolic blood pressure remained high (~200 mmHg), and ventricular hypertrophy remained unchanged. Ventricular arrhythmia inducibility (100 % inducible) and corrected QT interval (on ECG) did not change in HFpEF rats after diet modification. HFpEF rats with diet modification showed prolonged survival and reduced ventricular fibrosis (Masson’s trichrome staining) compared to high-salt-fed HFpEF rats. Hence, the modification of diet (from high-salt to normal-salt diet) reversed HFpEF phenotypes without affecting blood pressure or ventricular hypertrophy.

## 1. Introduction

Heart failure with preserved ejection fraction (HFpEF) accounts for approximately 50 % of all heart failure cases [1]. Although previously seen as a simple hemodynamic disorder of diastolic function [2], further research has shown the complexity of the disease as the pathology spans multiple organ systems [3]. Tens of clinical trials with medications adapted from HF with reduced EF (HFrEF), did not show the same survival benefit [4–7]. Despite rigorous research and numerous clinical trials, the definitive and comprehensive mechanisms of HFpEF are still illusive. Ventricular arrhythmias are common in patients with HFpEF [8], and sudden death is the most common mode of death in patients with HFpEF [9]. Hypertension has been well established as one of the main culprits that cause ventricular hypertrophy and subsequent diastolic dysfunction [10]. However, recent research overturned this dogma and verified that systemic inflammation and subsequent fibrosis are more important in the development of HFpEF [11,12].

Hypertension is the most common co-morbidity (~90 %) in patients with HFpEF [6,7]. Thus, hypertension has been used in many animal models to induce HFpEF [13]. Dahl salt-sensitive (DSS) rats develop hypertension when fed a high-salt (8 % NaCl) diet [14]. Subsequent ventricular hypertrophy and diastolic dysfunction are the cornerstone mechanisms of HFpEF [14]. While normal-salt diet does not induce HFpEF, the role of diet modification once HFpEF is established, have not been investigated. Here, we sought to investigate the role of high-salt vs. normal-salt diet on HFpEF progression using the DSS rat model of HFpEF.

## 2. Materials and methods

### 2.1. Animal model of HFpEF

**Fig. 1.**
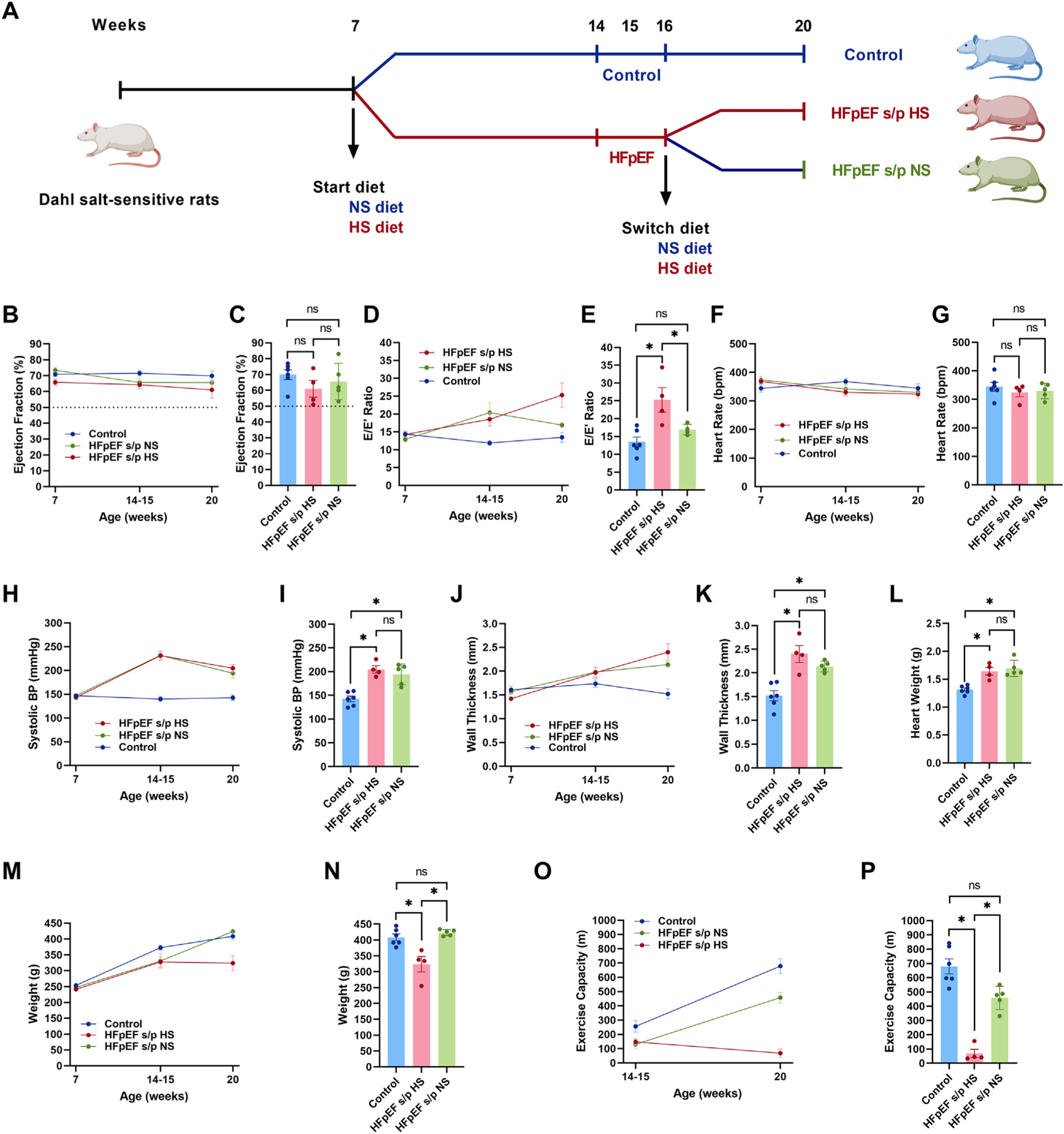
Diet switch reversed diastolic dysfunction in HFpEF. A. Experimental design of the project. B. Trend of EF in three groups of rats at each time point. C. EF at 20 weeks of age did not differ between HFpEF s/p HS and HFpEF s/p NS rats. D. Trend of E/E′ ratio in three groups of rats. E. E/E′ ratio was significantly reduced in HFpEF s/p NS rats as compared to HFpEF s/p HS rats (*p* = 0.0484) at 20 weeks of age. F. Trend of heart rate in three groups of rats. G. There was no difference in heart rate between HFpEF s/p HS and HFpEF s/p NS rats at 20 weeks of age. H. Trend of systolic blood pressure in three groups of rats at three time points. I. There was no difference in systolic blood pressure between HFpEF s/p HS and HFpEF s/p NS rats at 20 weeks of age. J. Trend of ventricular wall thickness in three groups of rats. K. No significant difference in ventricular hypertrophy was noted between HFpEF s/p HS and HFpEF s/p NS rats at 20 weeks of age. L. There was no difference in heart weight between HFpEF s/p HS and HFpEF s/p NS rats at 20 weeks of age. M. Trend of body weight in three groups of rats. N. Significant increase of body weight was observed in HFpEF s/p NS rats as compared to HFpEF s/p HS rats (*p* = 0.0009) at 20 weeks of age. O. Trend of exercise capacity in three groups of rats at two time points. P. Exercise capacity was markedly improved after diet switch in HFpEF rats (*p* = 0.0002) at 20 weeks of age. *N* = 6 rats for control, *N* = 4 rats for HFpEF s/p HS, and *N* = 5 rats for HFpEF s/p NS. ANOVA was used for the statistical analyses (* denotes *p* value <0.05 and ns denotes non-significant). Data are presented as means ± standard error of the mean.

Male DSS rats were received at 6 weeks old (Charles River Laboratories, MA) and were assigned to be fed either a normal-salt (NS) diet (0.3 % NaCl, AIN-76A) or a high-salt (HS) diet (8 % NaCl, AIN-76A with added 8 % NaCl, Research Diets, NJ) from 7 weeks of age (Fig. 1A). Rats were fed ad libitum. Prior to the implementation of the different diets, a rodent transthoracic echocardiogram was performed to establish a baseline systolic and diastolic function. A baseline non-invasive blood pressure measurement was also performed before the initiation of the diets. When the rats reached 14–15 weeks of age (when the rats are known to develop HFpEF), echocardiogram and blood pressure measurement were repeated. Once the HS-fed rats are confirmed to have diastolic dysfunction along with HF manifestations (weakness, weight loss, edema, reduced activity, and disturbed breathing, etc.), the rats were diagnosed as HFpEF (Fig. 1A). Then the HFpEF rats were randomized to either continue the HS diet (HFpEF s/p HS) or switch diet to NS (HFpEF s/p NS) at 16 weeks of age (Fig. 1A). NS-fed DSS rats were continued to consume NS diets to serve as controls. The diets were continued ad libitum until the end of experiments (20 weeks of age). Echocardiogram and blood pressure measurements were repeated at the end of the experimental protocol.

### 2.2. Transthoracic echocardiogram

Rodent transthoracic echocardiogram (Vevo 3100, Fujifilm VisualSonics, Canada) was performed to measure systolic and diastolic function at 7, 14–15, and 20 weeks of age. All rats were induced with 5 % isoflurane and were kept sedated throughout the procedure with 2 % isoflurane. The rats were prepped by shaving the thoracic region and removing the fur for clear imaging. The parasternal short-axis view of the left ventricle was visualized through the M-mode on the echocardiogram to measure EF. The papillary muscles were used as a landmark for consistency purposes. The rats were then placed on their left side and the apical 4-chamber view was achieved in order to assess the diastolic function. The pulse-wave Doppler mode was used to measure the E (Early filling) and the A (Atrial filling) of the left ventricle through the mitral valve and to calculate the E/A ratio. The tissue Doppler mode was used on the septal corner of the mitral annulus to measure the E′ wave which was used to calculate the E/E′ ratio. The measurements were performed twice and averaged to determine the exact systolic and diastolic functions of the rats.

### 2.3. Blood pressure measurement

Blood pressure was measured in the tail artery through a non-invasive transmission photoplethysmography machine (BP-2000, Visitech Systems, NC). The rats were not sedated during the measurement to avoid any sedation-induced blood pressure changes. The rats were placed in a small chamber with their tails placed through an occlusion cuff. Systolic and diastolic blood pressure and the heart rate were measured twice and averaged for each rat.

### 2.4. Rodent treadmill test

An Exer 3/6 rodent treadmill (Columbus Instruments, OH) was used to measure the exercise capacity of the rats at 14–15, and 20 weeks of age. Rats were placed on the treadmill with a 5-degree incline and a shock grid (activated at 0.15 mA with a frequency of one shock per second). Each session began with a 2-minute period of acclimation at a speed of 10 m/min and a deactivated shock grid. Then the shock grid was activated, and the speed of the treadmill began to accelerate at a rate of 1 m/min. After 5 consecutive shocks from the grid, the rats were removed from the treadmill, and the total distance traveled was recorded as the rats’ maximum functional capacity.

### 2.5. Electrocardiogram

Electrocardiogram (ECG) was recorded at 7, 14–15, and 20 weeks of age (Dual Bio Amps, AD Instruments, Australia). Each rat was induced with 5 % isoflurane and placed on the surgical bench (maintained with 2 % isoflurane) to measure the PR interval, QRS interval, QT interval, and RR interval. The measurements were repeated three times and averaged using LabChart 7 software (AD Instruments). Corrected QT (QTc) interval was calculated as QT interval (milliseconds) divided by the square root of RR interval (seconds) [15].

### 2.6. Programmed electrical stimulation

The rats were initially sedated with 5 % isoflurane and intubated for mechanical ventilation. A steady flow of 2 % isoflurane was maintained during the entire duration of the procedure. The core body temperature was maintained between 36 °C to 38° via a heating lamp and was monitored through a rectal probe. The heart was exposed by a left-sided thoracotomy, and the pericardial sac was opened so that an electrode could be placed in the apex of the left ventricle. The grounding electrode was placed into the chest wall. An electronic simulator (PowerLab, AD Instruments) was used to perform a programmed electrical stimulation using the standard protocol. A series of 10 stimuli of S1 (5 V, 1-millisecond pulse width, and 100-millisecond interval) was delivered through the probes. This series was followed by an extra-stimulus (S2; 5 V and 1 millisecond pulse width) starting at a coupling interval of 80 milliseconds that was shortened by 1 millisecond intervals until the effective refractory period was achieved. If ventricular arrhythmias were not induced, a second extra-stimulus (S3) was delivered from the last effective refractory period, and the coupling intervals were again shortened by 1 millisecond intervals until the effective refractory period was achieved. A third extra-stimulus (S4) was delivered in the same manner as S3 until the effective refractory period was achieved. If the rat failed to develop ventricular arrhythmias with this set of stimuli, the animal was deemed non-inducible. In the rats were inducible, the stimulations were repeated to confirm the reproducibility of ventricular arrhythmias.

### 2.7. Organ collection

After end-point experiments at 20 weeks of age, blood was withdrawn from tail vein and the rats were euthanized under 5 % isoflurane per protocol. The lungs, liver, and heart were collected and weighed. The heart was then placed in conical tubes containing 30 mL of 1:10 formalin.

### 2.8. B-type natriuretic peptide and comprehensive metabolic panel

Blood was centrifuged to collect serum. Serum B-type natriuretic peptide (BNP) was measured with enzyme-linked immunosorbent assay kit (RatBiotech, GA). The measurement was repeated twice and averaged. Collected serum was sent to the animal laboratory (Antech Diagnostics, CA) for comprehensive metabolic panel. Serum cholesterol, triglyceride, protein, albumin, AST, ALT and creatinine were measured.

### 2.9. Fibrosis measurement

The excised heart was fixed with 1:10 formalin for 24–48 h and stored in 70 % ethanol. The heart tissue was embedded in paraffin and sectioned in a short-axis configuration. The midventricular heart tissue was stained with Masson’s trichrome staining to visualize fibrosis. Each cross section was scanned and digitally processed through ImageJ software. The fractional ventricular fibrosis was calculated by dividing the number of blue pixels by the total number of pixels visualized.

### 2.10. Statistics

The differences between the groups in continuous data were tested with ANOVA, followed by a Tukey’s test to adjust for multiple testing. Residuals were inspected to confirm appropriateness of parametric testing. The differences in proportions were tested with exact Fisher’s test. Log-rank tests were used to analyze overall survival time. The data are presented as means ± standard error of the mean. The differences were considered significant when two-tailed *p*-values <0.05. Analysis performed with GraphPad Prism software (v9.3.1).

### 2.11. Study approval

All the rodent protocols and experiments were approved by the Cedars-Sinai Medical Center Institutional Animal Care and Use Committee.

## 3. Results

### 3.1. Diet switch reversed diastolic dysfunction in HFpEF

Systolic function as measured with EF by echocardiogram remained preserved in all three groups at the three time points (7, 14–15 and 20 weeks, Fig. 1B). The EF at 20 weeks of age did not differ between the three groups (Fig. 1C). Diastolic function as measured by the E/E′ ratio continued to deteriorate in HFpEF s/p HS while it improved in HFpEF s/p NS (Fig. 1D). The comparison of E/E′ ratio at 20 weeks was significant between the HFpEF s/p HS and HFpEF s/p NS rats (reversal of diastolic dysfunction, Fig. 1E). Heart rate during the echocardiogram was not different among the three groups at the three time points (control, HFpEF s/p HS, and HFpEF s/p NS, Fig. 1F–G). Systolic blood pressure was increased in HFpEF rats compared to the control rats at 14–15 weeks of age, and the diet switch did not reduce systolic blood pressure in HFpEF rats (Fig. 1H). Systolic blood pressure at 20 weeks of age was not different between the HFpEF s/p HS and HFpEF s/p NS rats (Fig. 1I). Ventricular wall thickness measured by echocardiogram was increased in HFpEF rats at 14–15 weeks of age but did not reduce after the diet switch (Fig. 1J). Ventricular hypertrophy was not different between the HFpEF s/p HS and HFpEF s/p NS rats at 20 weeks of age (Fig. 1K). Heart weight was increased in HFpEF rats compared to the control rats but did not change after the diet switch (Fig. 1L). Body weight was reduced in HFpEF rats at 14–15 weeks of age compared to the control rats, but the weight increased back to normal after diet switch (Fig. 1M). The body weight of the HFpEF s/p NS rats was markedly increased when compared to the HFpEF s/p HS rats (Fig. 1N). Exercise capacity was reduced in the HFpEF rats at 14–15 weeks of age but the diet modification markedly improved exercise capacity in the HFpEF rats (Fig. 1O). Exercise capacity was markedly improved in the HFpEF s/p NS rats at 20 weeks of age when compared to the HFpEF s/p HS rats (Fig. 1P). In summary, the diet switch from a HS diet to a NS diet in HFpEF rats reversed diastolic dysfunction, increased body weight, and improved exercise capacity without affecting blood pressure and ventricular hypertrophy.

### 3.2. Diet modification did not affect ventricular arrhythmias and electrical remodeling

**Fig. 2.**
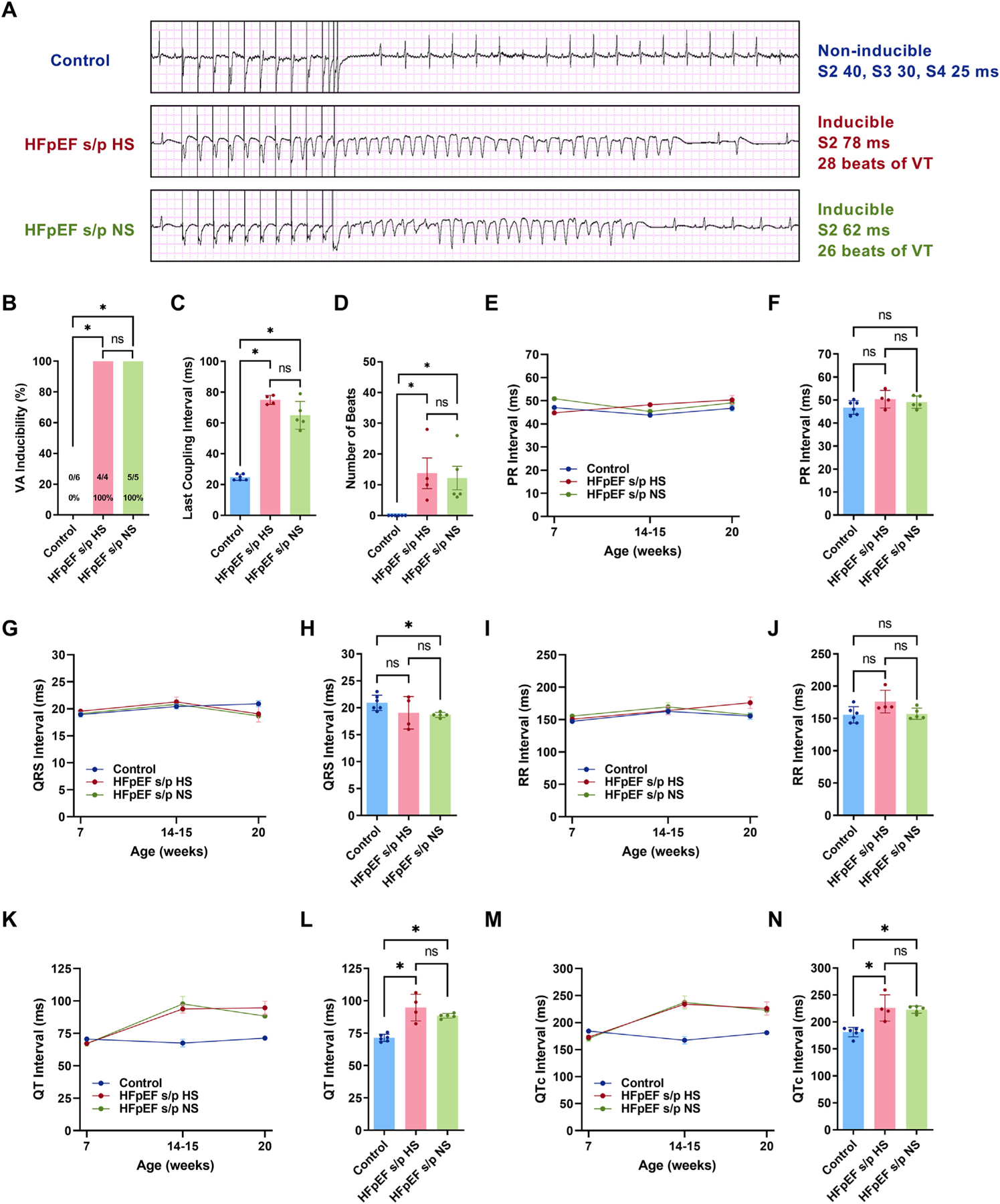
Diet modification did not affect ventricular arrhythmias and electrical remodeling. Programmed electrical stimulation tracings in three representative rats from each group. B. Ventricular arrhythmias inducibility did not change after diet switch in HFpEF rats. C. Last stimulus coupling interval was not altered after diet switch in HFpEF rats. D. The number of ventricular arrhythmias beats were not different between HFpEF s/p HS and HFpEF s/p NS rats. E. Trend of PR interval in three groups of rats at each time pint. F. There was no difference in PR interval between HFpEF s/p HS and HFpEF s/p NS rats at 20 weeks of age. G. Trend of QRS interval in three groups of rats. H. QRS interval was not altered after diet switch at 20 weeks of age. I. Trend RR interval in three groups of rats. J. There was no significant change in RR interval between HFpEF s/p HS and HFpEF s/p NS rats at 20 weeks of age. K. Trend of QT interval in three groups of rats. L. QT interval was not changed after diet switch in HFpEF rats at 20 weeks of age. M. Trend of QTc interval in three groups of rats. N. QTc interval was not altered in HFpEF rats after diet switch. *N* = 6 rats for control, N = 4 rats for HFpEF s/p HS, and N = 5 rats for HFpEF s/p NS. Exact Fisher’s test and ANOVA were used for the statistical analyses (* denotes *p* value <0.05 and ns denotes non-significant). Data are presented as means ± standard error of the mean.

Programmed electrical stimulation was applied to induce ventricular arrhythmias in the control and the HFpEF rats at 20 weeks of age (Fig. 2A). All of the rats in the HFpEF s/p HS and the HFpEF s/p NS groups induced ventricular arrhythmias while none of the control rats induced ventricular arrhythmias (Fig. 2B). There was no difference in the last coupling interval between the HFpEF s/p HS and HFpEF s/p NS rats (Fig. 2C). The duration of ventricular arrhythmias was similar between the HFpEF s/p HS and HFpEF s/p NS rats (Fig. 2D). ECG was analyzed to look at electrical remodeling. The PR interval did not show any meaningful trend in the three groups of rats at the three time points (Fig. 2E). The PR interval showed no differences between the HFpEF s/p HS and HFpEF s/p NS rats at 20 weeks of age (Fig. 2F). The QRS interval did not change between the three groups of rats (Fig. 2G). The QRS interval was similar between the HFpEF s/p HS and HFpEF s/p NS rats at 20 weeks of age (Fig. 2H). The RR interval did not change in the three groups of rats at the three time points (Fig. 2I). The RR interval was comparable between the HFpEF s/p HS and HFpEF s/p NS rats at 20 weeks of age (Fig. 2J). The QT interval was increased in HFpEF rats at 14–15 weeks of age, and the diet switch did not impact on the QT interval in HFpEF rats at 20 weeks of age (Fig. 2K). The QT interval did not change at 20 weeks of age between the HFpEF s/p HS and HFpEF s/p NS rats (Fig. 2L). The QTc interval showed a similar increase at 14–15 weeks of age in HFpEF rats (Fig. 2M) with no changes to the QTc interval at 20 weeks of age after the diet switch in HFpEF rats (Fig. 2N). In summary, the diet switch from HS to NS diet in HFpEF rats did not reduce ventricular arrhythmias, inducibility, or duration, and it had no impact on electrical remodeling.

### 3.3. Diet switch improved survival of HFpEF rats and reduced pulmonary and liver congestion

**Fig. 3.**
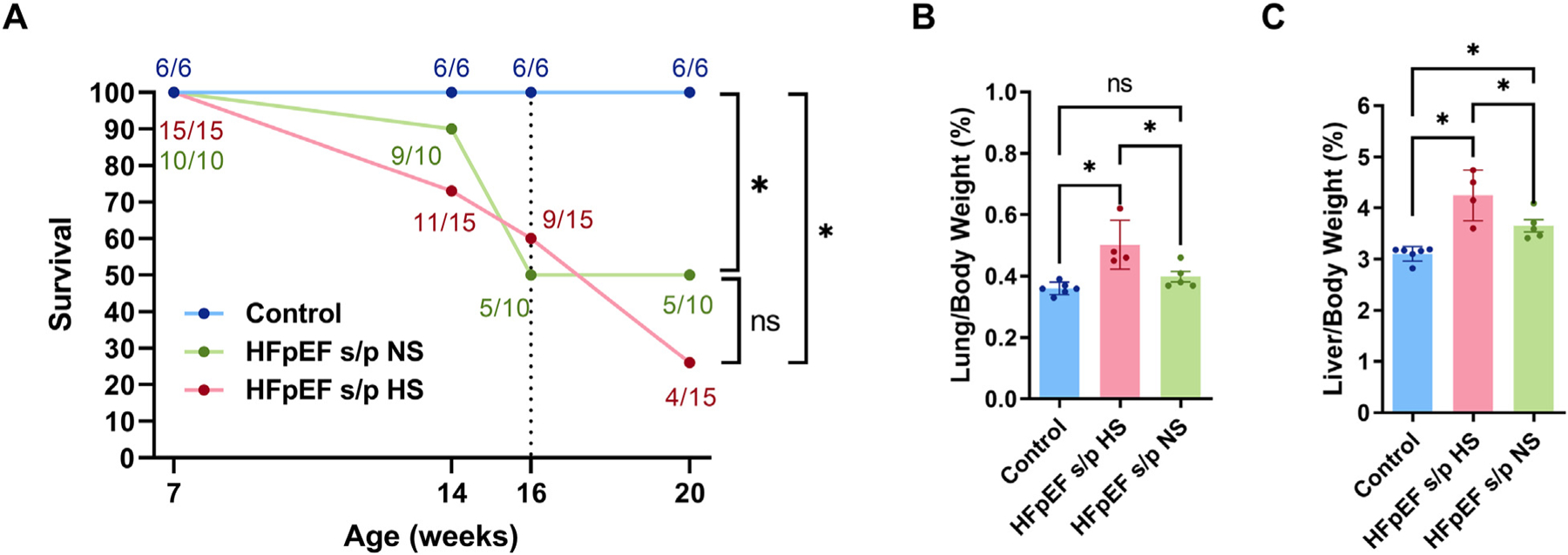
Diet switch improved survival of HFpEF rats and reduced pulmonary and liver congestion. A. Survival analysis of three groups of rats. Although the survival was different between HFpEF s/p HS and HFpEF s/p NS rats between 16 and 20 weeks (5/9 = 55 % mortality in HFpEF s/p HS rats vs. 0/5 = 0 % mortality in HFpEF s/p NS rats), it did not reach statistical significance due to low number of rats. B. Pulmonary congestion was significantly reduced in HFpEF s/p HS rats as compared to HFpEF s/p NS rats (*p* = 0.0162). C. Liver congestion was also significantly reduced in HFpEF s/p HS rats compared to HFpEF s/p NS rats (*p* = 0.0336). *N* = 6 rats for control, *N* = 15 rats for HFpEF s/p HS, and *N* = 10 rats for HFpEF s/p NS. Log-rank tests and ANOVA were used for the statistical analyses (* denotes *p* value <0.05 and ns denotes non-significant). Data are presented as means ± standard error of the mean.

The survival of the three groups of rats was checked at 14, 16 and 20 weeks of age (Fig. 3A). HFpEF rats are known to have increased mortality as HFpEF progresses. By 16 weeks of age, the HFpEF rats showed 40–50 % of mortality while none of the control rats died. The HFpEF s/p HS rats showed 56 % mortality from 16 weeks of age to 20 weeks of age (5/9 = 56 %) while none of the HFpEF s/p NS rats died during the same period (0/5 = 0 %). Lung/body weight showed reduction of pulmonary congestion in HFpEF s/p NS rats at 20 weeks of age (Fig. 3B). A similar reduction was noted in liver congestion as measured by the liver/body weight ratio in the HFpEF s/p NS rats (Fig. 3C). In summary, the diet switch to NS improved survival of the HFpEF rats and reduced pulmonary and liver congestion.

### 3.4. Diet switch reversed serum markers of HF

**Fig. 4.**
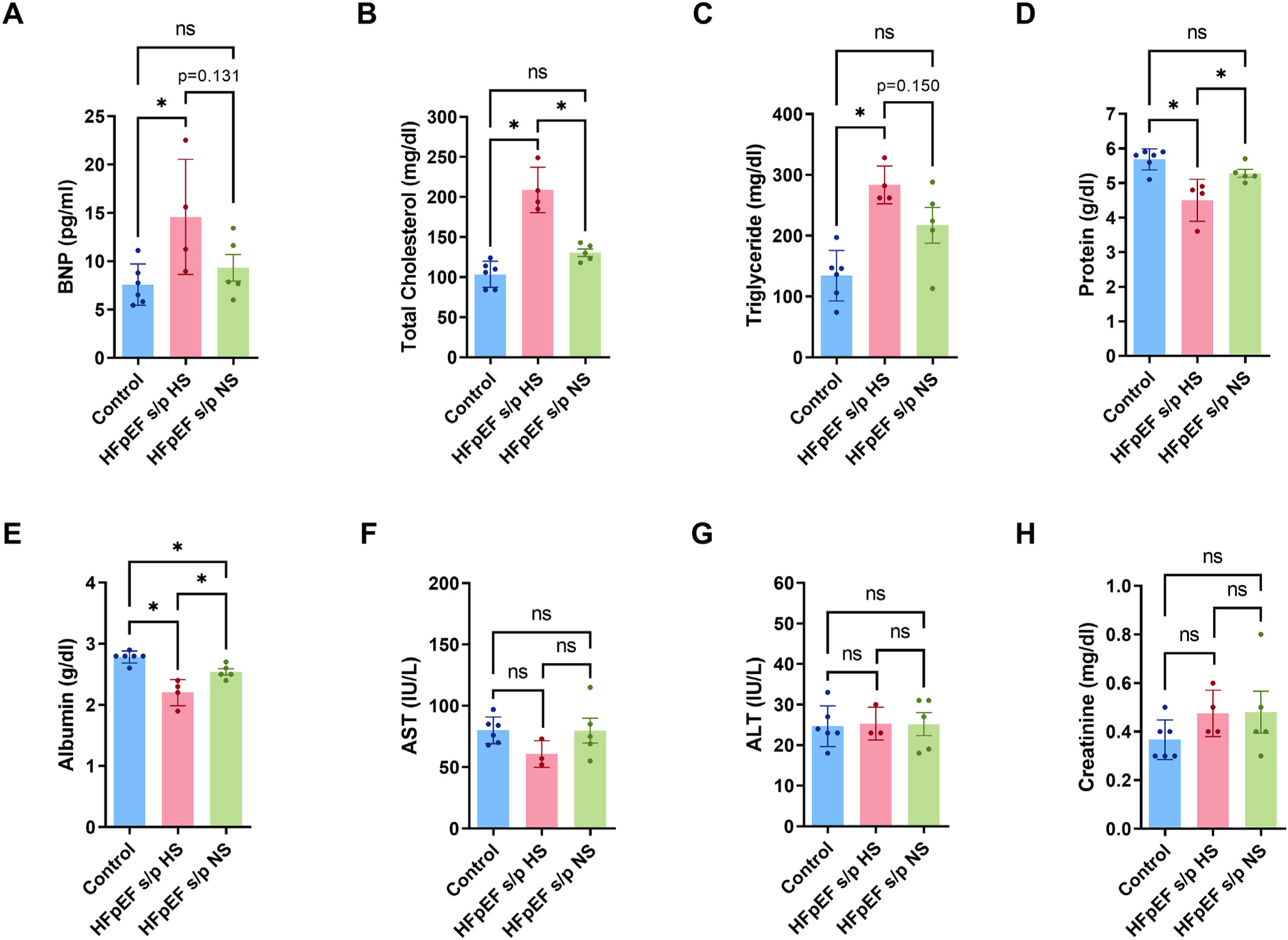
Diet switch reversed serum markers of HF. A. Serum BNP level was increased in HFpEF rats but showed strong trend of reduction in HFpEF s/p NS rats (*p* = 0.131). B. Serum total cholesterol was significantly reduced in HFpEF s/p NS rats as compared to HFpEF s/p HS rats (*p* = 0.0001). C. Serum triglyceride was increased in HFpEF rats and showed strong trend of reduction in diet switch group (*p* = 0.150). D. Serum protein was reduced in HFpEF rats and diet switch increased serum protein levels in HFpEF rats (*p* = 0.0291). E. Serum albumin was significantly increased in HFpEF s/p NS rats as compared to HFpEF s/p HS rats (*p* = 0.0098). F-G. Serum AST and ALT levels did not change between control and HFpEF rats. H. Serum creatinine level did not change between control and HFpEF rats. N = 6 rats for control, *N* = 4 rats for HFpEF s/p HS, and *N* = 5 rats for HFpEF s/p NS. ANOVA was used for the statistical analyses (* denotes p value <0.05 and ns denotes non-significant). Data are presented as means ± standard error of the mean.

The serum BNP was increased in the HFpEF rats but the diet switch showed a strong trend of reducing the serum BNP (Fig. 4A). Total cholesterol in the serum was increased in the HFpEF rats but was reduced after diet switch to a NS diet (Fig. 4B). The serum triglyceride was increased in the HFpEF rats but showed strong trend of reduction in HFpEF s/p NS rats (Fig. 4C). Serum protein and albumin was reduced in the HFpEF rats but increase after the diet switch to NS (Fig. 4D–E). The liver enzymes (AST and ALT) and creatinine were not altered in three groups of rats (Fig. 4F–H). In summary, the diet switch reversed serum markers of HF.

### 3.5. Diet switch reduced ventricular fibrosis

**Fig. 5.**
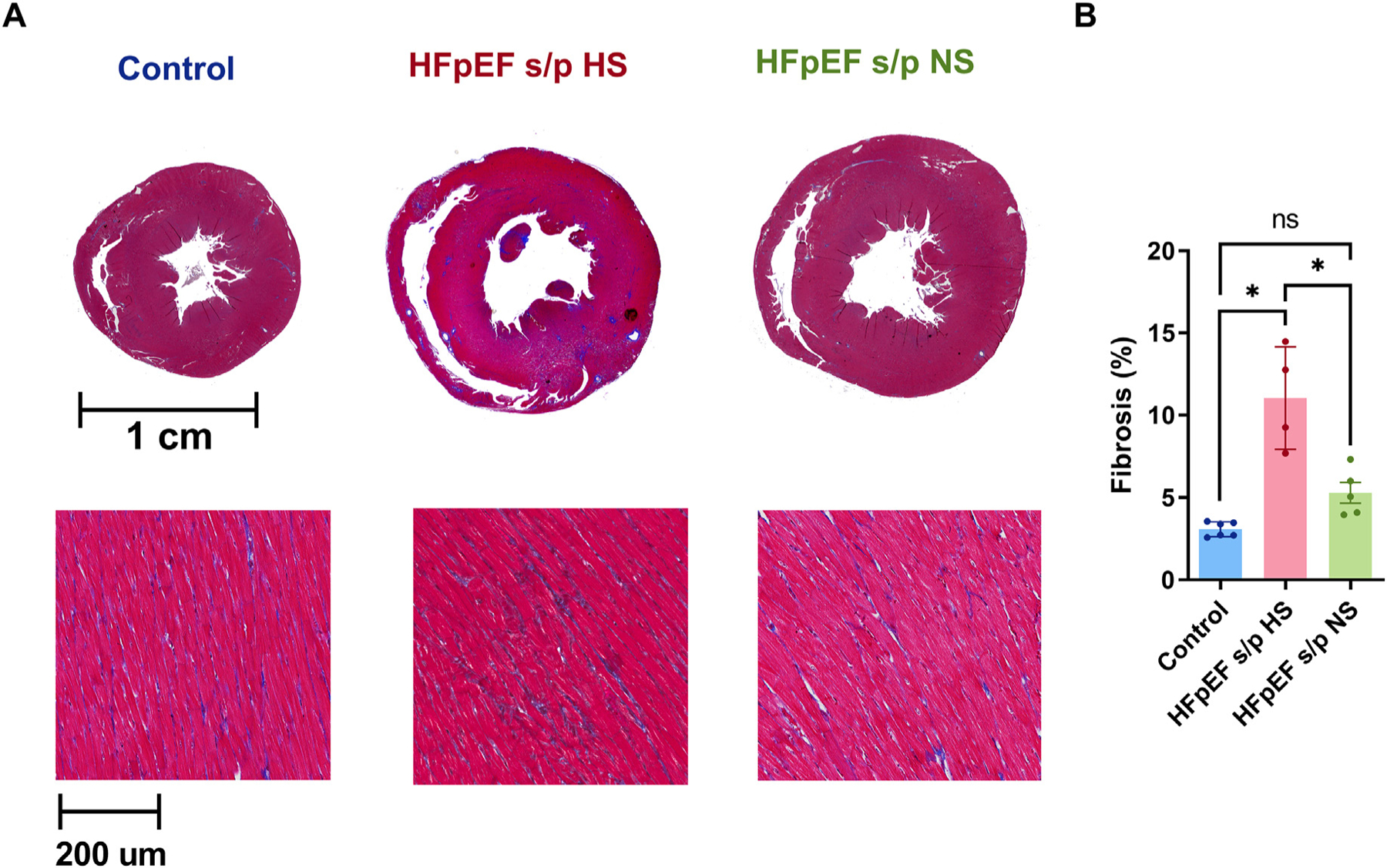
Diet switch reduced ventricular fibrosis. A. Representative Masson’s trichrome stating of control and HFpEF hearts at 20 weeks of age. B. Ventricular fibrosis was significantly reduced in HFpEF s/p NS rats as compared to HFpEF s/p HS rats (*p* = 0.0011). N = 6 rats for control, N = 4 rats for HFpEF s/p HS, and N = 5 rats for HFpEF s/p NS. ANOVA was used for the statistical analyses (* denotes p value <0.05 and ns denotes non-significant). Data are presented as means ± standard error of the mean.

Ventricular fibrosis was measured with Masson’s trichrome staining (Fig. 5A). The degree of ventricular fibrosis was reduced in the HFpEF s/p NS rats when compared to the HFpEF s/p HS rats (Fig. 5B).

## 4. Discussion

In the Dahl salt-sensitive rat model, a high-salt diet is imperative to induce HFpEF phenotypes. A high-salt diet induces hypertension, which can subsequently cause ventricular hypertrophy and diastolic dysfunction [14]. The continuation of a high-salt diet exacerbates the progression of HFpEF and renders the rats to expire by 20–24 weeks of age [16]. Here, we have shown that diet modification (from high-salt to normal-salt diet) in high-salt-fed HFpEF rats mitigated HFpEF phenotypes: reversed diastolic function, increased body weight, improved exercise capacity, reduced pulmonary and liver congestions, prolonged survival, reduced HF markers, and reduced ventricular fibrosis. However, blood pressure and ventricular hypertrophy did not alter after the diet modification. Hence, in this high-salt diet-based HFpEF rat model, the diet switch reversed HFpEF phenotypes without altering blood pressure or ventricular hypertrophy.

Contrary to the reversal of HFpEF phenotypes after the diet switch, the electrophysiological properties of HFpEF rats, i.e. inducible ventricular arrhythmias and prolonged QTc interval, did not change. Delayed repolarization manifested as prolonged QTc interval is an imperative substrate to induce ventricular arrhythmias in rats with HFpEF [14]. Action potential duration prolongation along with increased action potential heterogeneity renders cardiomyocytes electrically unstable [17]. These adverse electrical remodeling, when combined with premature ventricular contraction, triggers ventricular arrhythmias [15]. While it is surprising to observe that QTc interval did not shorten when most of the HFpEF phenotypes reverse, it is suggestive of the underlying mechanisms of ventricular arrhythmias in rats with HFpEF. One explanation is that once QTc is prolonged, i.e. once electrical remodeling occurs in HFpEF, it is resistant to the benefits caused by diet modification. Another possible explanation is that ventricular hypertrophy might underlie delayed repolarization since the diet switch did not reduce ventricular hypertrophy.

Our findings once again confirmed the emerging hypothesis of HFpEF development. In the traditional model of HFpEF pathogenesis, hypertension, and subsequent hypertrophy were the main culprit of the disease [10]. However, this dogma has been replaced by a new emerging concept; systemic inflammation and resultant ventricular fibrosis are believed to medicate the pathogenesis of HFpEF [10]. This emerging new concept has once again been supported by the lack of improvement of systolic blood pressure, but the reversal of several HFpEF phenotypes. The key for the development of HFpEF is the ventricular fibrosis, and not necessarily the hypertension or the ventricular hypertrophy.

Some limitations of the experiment must be acknowledged. First, DSS rats are bred to have a hypersensitivity towards salt, a sensitivity that may change the fundamental nature of how salt may realistically affect the development, progression, and treatment of HFpEF. Secondly, the model does not take into consideration other comorbidities that are commonly found in patients diagnosed with HFpEF such as obesity or diabetes. Thirdly, the experiments were performed only on male DSS rats, and although the sex differences in HFpEF progression after the diet switch is intriguing, this was out of our initial scope of investigation. Fourthly, we have only observed phenotypic changes of HFpEF rats after the diet modification, and have not performed any mechanistic investigation. Future research is needed to further probe the mechanisms of diet modification on HFpEF progression. Finally, it is clear that further research must be conducted in order to investigate the inflammatory response’s role in HFpEF, and their contribution to ventricular arrhythmias.
